# Supplementary material for: "Factors associated with provider unwillingness to perform induced abortion in Argentina: A cross-sectional study in four provinces following the legalization of abortion on request"
Source: PLoS One. 2023 Oct 4;18(10):e0292130. doi: 10.1371/journal.pone.0292130 (PMC10550142; doi:10.1371/journal.pone.0292130)
Supplement: S1 Table — (DOCX) [file pone.0292130.s002.docx]

## Supplementary Table 1: Associated factors with unwillingness to performing induced abortions

|  | **To save a woman’s life** | | | | **To preserve a woman’s health** | | | | **In case of rape** | | | | **On request** | | | |
| --- | --- | --- | --- | --- | --- | --- | --- | --- | --- | --- | --- | --- | --- | --- | --- | --- |
| **Variables** | **n/N** | **%*** | **OR (95% CI)** | **p-value** | **n/N** | **%*** | **OR (95% CI)** | **p-value** | **n/N** | **%*** | **OR (95% CI)** | **p-value** | **n/N** | **%*** | **OR (95% CI)** | **p-value** |
| **Province** |  |  |  |  |  |  |  |  |  |  |  |  |  |  |  |  |
| 1 | 5/25 | 20.0 | 1 | 0.006 | 10/21 | 47.6 | 1 | 0.018 | 9/26 | 34.6 | 1 | 0.009 | 12/25 | 48.0 | 1 | 0.143 |
| 2 | 22/34 | 64.7 | 6.2 (2.0;22.1) |  | 20/23 | 87.0 | 6.4 (1.7;30.0) |  | 20/27 | 74.1 | 5.8 (1.9;20.2) |  | 20/26 | 76.9 | 3.2 (1.0;10.8) |  |
| 3 | 0/9 | 0.0 | - |  | 0/9 | 0.0 | - |  | 0/9 | 0.0 | - |  | 0/8 | 0.0 | - |  |
| 4 | 6/14 | 42.9 | 2.8 (0.7;12.0) |  | 4/8 | 50.0 | 1.4 (0.3;7.7) |  | 6/14 | 42.9 | 2.2 (0.5;9.0) |  | 6/12 | 50.0% | 1.4 (0.3;6.4) |  |
| **Facility type: Primary Care** | | | |  |  |  |  |  |  |  |  |  |  |  |  |  |
| Yes | 5/15 | 33.3 | 0.6 (0.2;1.9) | 0.429 | 3/11 | 27.3 | 0.2 (0.1;0.9) | 0.033 | 4/16 | 25.0 | 0.3 (0.1;1.0) | 0.052 | 6/17 | 35.3 | 0.3 (0.1;1.0) | 0.052 |
| No | 28/67 | 41.8 | 1 |  | 31/50 | 62.0 | 1 |  | 31/60 | 51.7 | 1 |  | 32/54 | 59.3 | 1 |  |
| **Facility type: Secondary Care** | | | |  |  |  |  |  |  |  |  |  |  |  |  |  |
| Yes | 6/16 | 37.5 | 0.8 (0.2;2.3) | 0.650 | 6/15 | 40.0 | 0.4 (0.1;1.3) | 0.139 | 6/16 | 37.5 | 0.6 (0.2;1.9) | 0.430 | 8/16 | 50.0 | 0.7 (0.2;2.2) | 0.586 |
| No | 27/66 | 40.9 | 1 |  | 28/46 | 60.9 | 1 |  | 29/60 | 48.3 | 1 |  | 30/55 | 54.5 | 1 |  |
| **Facility type: Tertiary Care** | | | |  |  |  |  |  |  |  |  |  |  |  |  |  |
| Yes | 23/56 | 41.1 | 1.3 (0.5;3.4) | 0.591 | 25/38 | 65.8 | 3.1 (1.1;9.3) | 0.033 | 26/49 | 53.1 | 2.2 (0.9;6.2) | 0.103 | 25/43 | 58.1 | 1.9 (0.7;5.0) | 0.194 |
| No | 10/26 | 38.5 | 1 |  | 9/23 | 39.1 | 1 |  | 9/27 | 33.3 | 1 |  | 13/28 | 46.4 | 1 |  |
| **Age (years)** |  |  |  |  |  |  |  |  |  |  |  |  |  |  |  |  |
| <30 | 2/6 | 33.3 | 1.1 (0.2;5.7) | 0.118 | 1/4 | 25.0 | 0.4 (0.0;2.9) | 0.158 | 1/6 | 16.7 | 0.5 (0.1;3.2) | 0.008 | 1/5 | 20.0 | 0.4 (0.0;2.3) | 0.010 |
| >=30 and <45 | 15/47 | 31.9 | 1 |  | 17/35 | 48.6 | 1 |  | 16/46 | 34.8 | 1 |  | 18/42 | 42.9 | 1 |  |
| >=45 and <=60 | 12/23 | 52.2 | 3.0 (1.0;8.9) |  | 13/18 | 72.2 | 2.5 (0.8;8.6) |  | 16/21 | 76.2 | 4.8 (1.6;16.3) |  | 17/21 | 81.0 | 4.5 (1.4;16.8) |  |
| **Gender** |  |  |  |  |  |  |  |  |  |  |  |  |  |  |  |  |
| Male | 11/27 | 40.7 | 1.1 (0.4;2.9) | 0.808 | 12/16 | 75.0 | 3.0 (0.9;11.5) | 0.065 | 11/21 | 52.4 | 1.5 (0.5;4.2) | 0.452 | 11/20 | 55.0 | 1.2 (0.4;3.5) | 0.741 |
| Female | 21/54 | 38.9 | 1 |  | 20/43 | 46.5 | 1 |  | 22/53 | 41.5 | 1 |  | 25/49 | 51.0 | 1 |  |
| **Number of years in practice** | | | |  |  |  |  |  |  |  |  |  |  |  |  |  |
| <10 | 9/28 | 32.1 | 0.8 (0.3;2.2) | 0.475 | 8/19 | 42.1 | 0.7 (0.2;2.1) | 0.505 | 8/29 | 27.6 | 0.5 (0.2;1.4) | 0.092 | 8/27 | 29.6 | 0.4 (0.1;1.1) | 0.022 |
| >=10 and <20 | 13/34 | 38.2 | 1 |  | 15/27 | 55.6 | 1 |  | 15/31 | 48.4 | 1 |  | 16/27 | 59.3 | 1 |  |
| >=20 and <=42 | 7/16 | 43.8 | 1.8 (0.5;6.3) |  | 8/12 | 66.7 | 1.5 (0.4;6.4) |  | 9/13 | 69.2 | 2.1 (0.6;8.6) |  | 11/14 | 78.6 | 2.3 (0.6;10.7) |  |

*The proportion was calculated as the number of providers who were not willing to do the abortion and were included in that variable´s category divided by the number of providers that were included in that variable´s category.

The reference group is the one that has a “1” in the OR column.
