# Supplementary material for: "Factors associated with provider unwillingness to perform induced abortion in Argentina: A cross-sectional study in four provinces following the legalization of abortion on request"
Source: PLoS One. 2023 Oct 4;18(10):e0292130. doi: 10.1371/journal.pone.0292130 (PMC10550142; doi:10.1371/journal.pone.0292130)
Supplement: S2 Table — (DOCX) [file pone.0292130.s003.docx]

| **Variables** | **Proportion*** | % | **Unadjusted** | **p-value** | **Adjusted Odds^β^** | **p-value** |
| --- | --- | --- | --- | --- | --- | --- |
| **District** |  |  |  |  |  |  |
| 1 | 5/25 | 20.0 | 1 | 0.006 | 1 | 0.006 |
| 2 | 22/34 | 64.7 | 6.2 (2.0;22.1) |  | 6.2 (2.0;22.1) |  |
| 3** | 0/9 | 0.0 | - |  | - |  |
| 4 | 6/14 | 42.9 | 2.8 (0.7;12.0) |  | 2.8 (0.7;12.0) |  |
| **Facility type: Primary Care** |  |  |  |  |  |  |
| Yes | 5/15 | 33.3 | 0.6 (0.2;1.9) | 0.429 | - | - |
| No | 28/67 | 41.8 | 1 |  |  |  |
| **Facility type: Secondary Care** |  |  |  |  |  |  |
| Yes | 6/16 | 37.5 | 0.8 (0.2;2.3) | 0.650 | - | - |
| No | 27/66 | 40.9 | 1 |  |  |  |
| **Facility type: Tertiary Care** |  |  |  |  |  |  |
| Yes | 23/56 | 41.1 | 1.3 (0.5;3.4) | 0.591 | - | - |
| No | 10/26 | 38.5 | 1 |  |  |  |
| **Age (years)** |  |  |  |  |  |  |
| <30 | 2/6 | 33.3 | 1.1 (0.2;5.7) | 0.118 | - | - |
| >=30 and <45 | 15/47 | 31.9 | 1 |  |  |  |
| >=45 and <=60 | 12/23 | 52.2 | 3.0 (1.0;8.9) |  |  |  |
| **Gender** |  |  |  |  |  |  |
| Male | 11/27 | 40.7 | 1.1 (0.4;2.9) | 0.808 | - | - |
| Female | 21/54 | 38.9 | 1 |  |  |  |
| **Number of years in practice** |  |  |  |  |  |  |
| <10 | 9/28 | 32.1 | 1 | 0.475 | - | - |
| >=10 and <20 | 13/34 | 38.2 | 1.3 (0.5;3.7) |  |  |  |
| >=20 and <=42 | 7/16 | 43.8 | 2.2 (0.6;8.6) |  |  |  |
| *The proportion was calculated as the number of providers who were not willing to do the abortion and were included in that variable´s category divided by the number of providers that were included in that variable´s category.  **Providers with this answer were not included in the calculation of the odds ratio.  The reference group is referred with a “1” in the OR column. | | | | | | |

## **Supplementary Table 2. Associated factors with unwillingness to performing induced abortions to save a woman’s life.**
