# Supplementary material for: "Factors associated with provider unwillingness to perform induced abortion in Argentina: A cross-sectional study in four provinces following the legalization of abortion on request"
Source: PLoS One. 2023 Oct 4;18(10):e0292130. doi: 10.1371/journal.pone.0292130 (PMC10550142; doi:10.1371/journal.pone.0292130)
Supplement: S3 Table — (DOCX) [file pone.0292130.s004.docx]

**Supplementary Table 3. Associated factors with unwillingness to performing induced abortions to preserve a woman’s health**

| **Variables** | **Proportion*** | % | **Unadjusted** | **p-value** | **Adjusted Odds^β^** | **p-value** |
| --- | --- | --- | --- | --- | --- | --- |
| **District** |  |  |  |  |  |  |
| 1 | 10/21 | 47.6 | 1 | 0.018 | 1 | 0.014 |
| 2 | 20/23 | 87.0 | 6.4 (1.7;30.0) |  | 8.2 (1.9;46.5) |  |
| 3** | 0/9 | 0.0 | - |  | - |  |
| 4 | 4/8 | 50.0 | 1.4 (0.3;7.7) |  | 1.2 (0.2;7.7) |  |
| **Facility type: Primary Care** |  |  |  |  |  |  |
| Yes | 3/11 | 27.3 | 0.2 (0.1;0.9) | 0.033 | - | - |
| No | 31/50 | 62.0 | 1 |  |  |  |
| **Facility type: Secondary Care** |  |  |  |  |  |  |
| Yes | 6/15 | 40.0 | 0.4 (0.1;1.3) | 0.139 | - | - |
| No | 28/46 | 60.9 | 1 |  |  |  |
| **Facility type: Tertiary Care** |  |  |  |  |  |  |
| Yes | 25/38 | 65.8 | 3.1 (1.1;9.3) | 0.033 | 5.1 (1.4;23.3) | 0.010 |
| No | 9/23 | 39.1 | 1 |  | 1 |  |
| **Age (years)** |  |  |  |  |  |  |
| <30 | 1/4 | 25.0 | 0.4 (0.0;2.9) | 0.158 | - | - |
| >=30 and <45 | 17/35 | 48.6 | 1 |  |  |  |
| >=45 and <=60 | 13/18 | 72.2 | 2.5 (0.8;8.6) |  |  |  |
| **Gender** |  |  |  |  |  |  |
| Male | 12/16 | 75.0 | 3.0 (0.9;11.5) | 0.065 | - | - |
| Female | 20/43 | 46.5 | 1 |  |  |  |
| **Number of years in practice** |  |  |  |  |  |  |
| <10 | 8/19 | 42.1 | 1 | 0.505 | - | - |
| >=10 and <20 | 15/27 | 55.6 | 1.5 (0.5;5.1) |  |  |  |
| >=20 and <=42 | 8/12 | 66.7 | 2.3 (0.6;10.7) |  |  |  |
| *The proportion was calculated as the number of providers who were not willing to do the abortion and were included in that variable´s category divided by the number of providers that were included in that variable´s category.  **Providers with this answer were not included in the calculation of the odds ratio.  The reference group is referred with a “1” in the OR column.  β Adjusted OR obtained from a multivariate model in which the remained significant variables are included. | | | | | | |
