# Supplementary material for: "Factors associated with provider unwillingness to perform induced abortion in Argentina: A cross-sectional study in four provinces following the legalization of abortion on request"
Source: PLoS One. 2023 Oct 4;18(10):e0292130. doi: 10.1371/journal.pone.0292130 (PMC10550142; doi:10.1371/journal.pone.0292130)
Supplement: S4 Table — (DOCX) [file pone.0292130.s005.docx]

**Supplementary Table 4. Associated factors with unwillingness to performing induced abortions in case of rape**

| **Variables** | **Proportion*** | % | **Unadjusted** | **p-value** | **Adjusted Odds^β^** | **p-value** |
| --- | --- | --- | --- | --- | --- | --- |
| **District** |  |  |  |  |  |  |
| 1 | 9/26 | 34.6 | 1 | 0.009 | 1 | 0. 009 |
| 2 | 20/27 | 74.1 | 5.8 (1.9;20.2) |  | 5.8 (1.9;20.2) |  |
| 3** | 0/9 | 0.0 | - |  | - |  |
| 4 | 6/14 | 42.9 | 2.2 (0.5;9.0) |  | 2.2 (0.5;9.0) |  |
| **Facility type: Primary Care** |  |  |  |  |  |  |
| Yes | 4/16 | 25.0 | 0.3 (0.1;1.0) | 0.052 | - | - |
| No | 31/60 | 51.7 | 1 |  |  |  |
| **Facility type: Secondary Care** |  |  |  |  |  |  |
| Yes | 6/16 | 37.5 | 0.6 (0.2;1.9) | 0.430 | - | - |
| No | 29/60 | 48.3 | 1 |  |  |  |
| **Facility type: Tertiary Care** |  |  |  |  |  |  |
| Yes | 26/49 | 53.1 | 2.2 (0.9;6.2) | 0.103 | - | - |
| No | 9/27 | 33.3 | 1 |  |  |  |
| **Age (years)** |  |  |  |  |  |  |
| <30 | 1/6 | 16.7 | 0.5 (0.1;3.2) | 0.008 | - | - |
| >=30 and <45 | 16/46 | 34.8 | 1 |  |  |  |
| >=45 and <=60 | 16/21 | 76.2 | 4.8 (1.6;16.3) |  |  |  |
| **Gender** |  |  |  |  |  |  |
| Male | 11/21 | 52.4 | 1.5 (0.5;4.2) | 0.452 | - | - |
| Female | 22/53 | 41.5 | 1 |  |  |  |
| **Number of years in practice** |  |  |  |  |  |  |
| <10 | 8/29 | 27.6 | 1 | 0.092 | - | - |
| >=10 and <20 | 15/31 | 48.4 | 2.1 (0.7;6.3) |  |  |  |
| >=20 and <=42 | 9/13 | 69.2 | 4.3 (1.1;19.0) |  |  |  |
| *The proportion was calculated as the number of providers who were not willing to do the abortion and were included in that variable´s category divided by the number of providers that were included in that variable´s category.  **Providers with this answer were not included in the calculation of the odds ratio.  The reference group is referred with a “1” in the OR column.  β Adjusted OR obtained from a multivariate model in which the remained significant variables are included. | | | | | | |
