# Supplementary material for: "Factors associated with provider unwillingness to perform induced abortion in Argentina: A cross-sectional study in four provinces following the legalization of abortion on request"
Source: PLoS One. 2023 Oct 4;18(10):e0292130. doi: 10.1371/journal.pone.0292130 (PMC10550142; doi:10.1371/journal.pone.0292130)
Supplement: S5 Table — (DOCX) [file pone.0292130.s006.docx]

## **Supplementary Table 5. Associated factors with unwillingness to performing induced abortions on request**

| **Variables** | **Proportion*** | % | **Unadjusted** | **p-value** | **Adjusted Odds^β^** | **p-value** |
| --- | --- | --- | --- | --- | --- | --- |
| **District** |  |  |  |  |  |  |
| 1 | 12/25 | 48.0 | 1 | 0.143 | - | - |
| 2 | 20/26 | 76.9 | 3.2 (1.0;10.8) |  |  |  |
| 3** | 0/8 | 0.0 | - |  |  |  |
| 4 | 6/12 | 50.0 | 1.4 (0.3;6.4) |  |  |  |
| **Facility type: Primary Care** |  |  |  |  |  |  |
| Yes | 6/17 | 35.3 | 0.3 (0.1;1.0) | 0.052 | - | - |
| No | 32/54 | 59.3 | 1 |  |  |  |
| **Facility type: Secondary Care** |  |  |  |  |  |  |
| Yes | 8/16 | 50.0 | 0.7 (0.2;2.2) | 0.586 | - | - |
| No | 30/55 | 54.5 | 1 |  |  |  |
| **Facility type: Tertiary Care** |  |  |  |  |  |  |
| Yes | 25/43 | 58.1 | 1.9 (0.7;5.0) | 0.194 | - | - |
| No | 13/28 | 46.4 | 1 |  |  |  |
| **Age (years)** |  |  |  |  |  |  |
| <30 | 1/5 | 20.0 | 0.4 (0.0;2.3) | 0.010 | 0.4 (0.0;2.3) | 0.010 |
| >=30 and <45 | 18/42 | 42.9 | 1 |  | 1 |  |
| >=45 and <=60 | 17/21 | 81.0 | 4.5 (1.4;16.8) |  | 4.5 (1.4;16.8) |  |
| **Gender** |  |  |  |  |  |  |
| Male | 11/20 | 55.0 | 1.2 (0.4;3.5) | 0.741 | - | - |
| Female | 25/49 | 51.0 | 1 |  |  |  |
| **Number of years in practice^£^** |  |  |  |  |  |  |
| <10 | 8/27 | 29.6 | 1 | 0.022 | - | - |
| >=10 and <20 | 16/27 | 59.3 | 2.8 (0.9;8.8) |  |  |  |
| >=20 and <=42 | 11/14 | 78.6 | 6.4 (1.6;31.1) |  |  |  |
| *The proportion was calculated as the number of providers who were not willing to do the abortion and were included in that variable´s category divided by the number of providers that were included in that variable´s category.  **Providers with this answer were not included in the calculation of the odds ratio.  β Adjusted OR obtained from a multivariate model in which the remained significant variables are included.  **^£^**The variable was not included in the multivariate model due to the presence of collinearity with age  The reference group is referred with a “1” in the OR column. | | | | | | |
